# Calibration of dentists for Caries Management by Risk Assessment Research in a Practice Based Research Network - CAMBRA PBRN

**DOI:** 10.1186/s12903-017-0457-3

**Published:** 2018-01-04

**Authors:** Peter Rechmann, Bonnie Jue, William Santo, Beate M. T. Rechmann, John D. B. Featherstone

**Affiliations:** School of Dentistry, Department of Preventive and Restorative Dental Sciences, University of California, 707 Parnassus Avenue, San Francisco, CA 94019 USA

**Keywords:** Dental caries, Caries Management by Risk Assessment – CAMBRA, Practice Based Research Network – PBRN, Examiner calibration, DMFS index, ICDAS, Inter-examiner reliability

## Abstract

**Background:**

To prove that Caries Management by Risk Assessment (CAMBRA) can be successfully implemented in dental practices outside of the university setting, dentists in the San Francisco Bay Area (CA) were approached to participate in a Practice Based Research Network (PBRN) study. The overall goal of the CAMBRA-PBRN study was to recruit 30 dentists to perform a two-year study involving approximately 900 patients. Goal of the calibration study was to standardize and calibrate dentists potentially participating in the CAMBRA-PBRN study.

**Methods:**

To minimize inter-examiner variability in data collection, including classification of carious lesions and recording of existing restorations, participating dentists were trained and calibrated in accurate DMFS (decayed, missing, filled surfaces) charting. Dentists were also trained and calibrated to diagnose and differentiate between sound surfaces and non-cavitated caries lesions (International Caries Detection and Assessment - ICDAS scores 1 and 2) for posterior occlusal surfaces.

Thirty dentists were calibrated to a single gold standard examiner (BJ) during 6 calibration sessions, between 2011 and 2014. Kappa statistics were used to determine inter-examiner reliability on 13 or more patients, aged 12–63 (average age 38 ± 15 years), per examiner during each session, resulting in 94 patient encounters over the course of all 6 sessions. To participate in the main study, examiners needed to achieve a minimum required kappa of 0.75. During the calibration process, examiners scored between 1036 and 2220 tooth surfaces.

**Results:**

The kappa values (unweighted kappa**)** of the participating dentists compared to the gold standard examiner ranged from 0.75 to 0.90, with an average kappa of 0.84 ± 0.03. 90% of the examiners achieved overall kappa values above 0.8. However, separate reliability for assessment of non-cavitated lesions, as in other studies, was lower (0.55 ± 0.15). Multiple subcategories were evaluated. All dentists reached sufficient reliability values to proceed into the study; nevertheless, one dentist discontinued with the study due to scheduling conflicts.

**Conclusions:**

The high inter-examiner reliability results have shown that dentists who work in primarily non-research based practices can be effectively standardized and calibrated in data collection, based on specific guidelines created to anticipate potential research study scenarios.

## Background

During the last two decades, dental clinicians have increasingly accepted minimally invasive treatment concepts [1–3]. Decisions to perform invasive restorative treatment have been delayed and can be performed at a more advanced caries lesion stage [4, 5].

Assessing the patient’s caries risk and assigning individualized preventive, non-operative treatments may become more appropriate [6, 7]. The Caries Management By Risk Assessment (CAMBRA) randomized, prospective, and controlled clinical trial performed at the University of California at San Francisco (UCSF) showed that an intervention featuring combined antibacterial and fluoride therapy significantly reduced bacterial load. The trial suggested a reduced caries increment in adults who had at the start of the trial a high caries risk, presenting 1 to 7 cavitated teeth at the baseline examination [6].

The CAMBRA system and philosophy was developed in California following two consensus conferences [8, 9]. Considerable research has been published related to the validation of the systems. The CAMBRA caries risk assessment (CRA) system was substantiated in two outcomes studies [10, 11]. In 2571 patients that were categorized at baseline as low, moderate, high or extreme caries risk, new cavitated lesions, radiographic lesion penetration into dentin or approximal enamel lesions on X-rays were found in 24%, 39%, 69% and 88%, respectively, at a follow-up examination 16 ± 13 months. It should be noted that those patients at extreme or high caries risk had not received the appropriate preventive measures [10].

Recently, it has been shown once more that the baseline caries risk is strongly associated with future caries. After adjustment for other patient characteristics, the predictive validity of the multi-component caries risk assessment approach upon which CAMBRA is based was again confirmed [12].

The original randomized, prospective, controlled CAMBRA trial had been proven to be successful in a university setting. The validation papers were also based on data available from university records including follow-ups of patients visiting the student clinics.

The overall goal of practice based research networks is to enhance clinical practice by engaging dentists in studies that are directly pertinent to daily clinical practice [13]. With the intention to prove that Caries Management by Risk Assessment (CAMBRA) [6, 8] can be successfully implemented in dental practices outside of the university setting, dentists in the San Francisco Bay Area were approached to participate in a Practice Based Research Network (PBRN) study. With support from the California Dental Association (CDA), a new network named CAMBRA-PBRN was created. The goal of the CAMBRA-PBRN study was to recruit 30 dentists to perform a two-year study involving approximately 900 patients. After initial screening and treatment of all cavitated caries lesions, and assessment of caries risk, patients would be randomly assigned to either an active preventive intervention or a “standard of care” control preventive treatment. Newly formed caries lesions and changes in caries risk status would be monitored as outcomes for the main study.

Because of the need for the study dentists to score oral conditions of a given study subject in a consistent manner, they were trained and calibrated in a caries diagnostic training and calibration seminar/workshop ahead of the main study. These examiners registered to participate in this calibration with other staff members of their dental offices to assure comparability between offices and in relation to other former and potential new CAMBRA studies. The goal of this calibration study presented in this paper was to minimize inter-examiner variability in data collection, classification of carious lesions and recording of existing restorations, by standardizing dentists in examining the dental status with existing restorations and the caries status (sound, non-cavitated lesions as ICDAS 1, 2 (International Caries Detection and Assessment System) [14, 15], DMFS – Decayed, Missing, Filled Surfaces [16, 17]) of the dentition of subjects before entering the main study. Dentists who successfully completed the training and calibration sessions would then later implement the main two-year CAMBRA study in their offices.

## Methods

Dentists in the San Francisco Bay area were recruited through advertisements and personal phone calls to attend information meetings about the planned University of California at San Francisco (UCSF) Investigational Review Board (IRB) approved CAMBRA-PBRN study (main study IRB #10–02153). Of the approximately 7300 licensed dentists who received information through the CDA newsletter about the intended CAMBRA study, around 100 stated interest and attended the information sessions.

Several one-day information sessions were offered by the California Dental Association (CDA). During those meetings, the main study design and the involvement of the dentist, as well as, the requirements to participate in the study were explained to the attending dentists. A total of 30 dentists (13 female, 17 male) enrolled to participate in the study. Three dentists were employed at different Federally Qualified Health Centers (FQHC) and 27 owned their dental offices. All enrolled dentists were General Dentists and had graduated 15 or more years ago.

The recruited dentists attended one of 5 training workshops (3 in 2011 and 2 in 2014) and one of 6 calibration sessions (3 in 2011 and 3 in 2014). The time interval between training workshops and calibration sessions varied between 0.5 and 1 day. The number of dentists per calibration session ranged between 3 and 8 with an average of 5 ± 1.7. The UCSF Institutional Review Board (IRB) reviewed and approved procedures for this calibration (IRB #10–04804) and all participating dentists gave informed written consent.

Prior to the workshops, each dentist received information material explaining the purpose of the training program, protocols for the training and calibration, and written instructions for the clinical examination of patients/calibration subjects. A PowerPoint presentation reviewing this examination criteria and data recording instructions were also provided. Prior to these training and calibration meetings, none of the dentists were familiar with the ICDAS scoring system.

### Training and calibration procedures

The training and calibration program included: 1) Review of dental status examination criteria and protocol, 2) Review of a PowerPoint presentation depicting the dental status examination criteria, 3) Evaluation of trainees’ knowledge of the criteria, 4) Clinical Training with demonstration examinations, 5) Practice Examinations by practitioners, and 6) Examiner Calibration. While steps 1 through 5 occurred during the training portion of the program, step 6 was carried out at the final calibration part of the program.

#### 1 and 2) Review of dental status examination criteria and protocol & review of PowerPoint presentation depicting the criteria

The examiner-trainees were encouraged to review the caries scoring criteria and protocol at home and were also asked to review the PowerPoint presentation depicting the caries scoring/evaluation criteria. During the training and calibration meetings, the gold standard examiner (BJ) reviewed the training material again with the dentists in person prior to seeing any calibration subjects for the training and calibration sessions. The gold standard examiner has extensive experience with calibration programs involving other multi-disciplinary and multi-institutional teams, including the Early Childhood Caries Collaborating Centers (EC4) [18], comprised of Boston University’s Center for Research to Evaluate and Eliminate Dental Disparities (CREEDD), University of Colorado Denver’s Center for Native Oral Health Research (CNOHR), and University of California, San Francisco’s Center to Address Disparities in Children’s Oral Health (CANDO). During these calibration sessions in 2010–2015, led by specialists from the University of Iowa, she consistently exceeded the standard kappa score of 0.75, in comparison with the other gold standard examiners.

#### 3) Evaluation of trainees’ knowledge of the criteria

The examiner-trainees were informed before the in-person meetings that the effectiveness of the training program would be assessed by a brief written quiz on the criteria. Specifically, the quiz would cover the details of the criteria and proper scoring of findings. The examiner-trainees were informed that this knowledge quiz had to be successfully completed, with at least 80% of the questions answered correctly, prior to proceeding to the calibration. In the case of an examiner-trainee not successfully completing this quiz, he/she would have had to complete an additional review of the criteria and protocol, and reattempt the quiz until he/she successfully scored 80%. The second subsequent quiz may have contained different content from the one previously taken. For this study, all dentists completed the knowledge quiz successfully on the first attempt.

#### 4 and 5) Clinical training with demonstration examinations and practice examinations by practitioners

The clinical training session began with 2–3 full mouth demonstration cases during which the gold standard examiner demonstrated the protocols and highlighted elements of the caries criteria apparent in the calibration subjects’ cases. These demonstrations were followed by 3–4 full mouth or half-mouth practice examinations for each examiner-trainee (or fewer, if subjects could be examined by multiple examiners) to allow each dentist to become familiar with the protocol, scoring criteria, and working routine with a recorder. These practice examinations also allowed for consultation with and clarification from the gold standard examiner.

#### 6) Examiner calibration

##### Subject recruitment and screening for calibration

The CAMBRA-PBRN main study was most interested in measuring caries increment after all patients had been “restored to health” and all treatments for cavitated lesions had been completed before subjects were enrolled into the study. However the examiners were required to assess demineralized, non-cavitated lesions that did not necessarily need restorations. Since the examiner calibration was meant to test examiners’ application of caries criteria, they were calibrated on all levels of disease presentation, including no disease.

Subjects (patient participants) enrolled in the calibration were required to fulfill inclusion and exclusion criteria in order to closely match the characteristics of the anticipated study sample. The subjects were recruited from UCSF’s School of Dentistry predoctoral student clinics by approaching them through their assigned student doctors.

Inclusion Criteria for the calibration:

- Be between 12 and 65 years of age;
- Have at least seven natural teeth remaining;
- Be in good health;
- Be of either gender;
- Have an understanding of the study (calibration);
- Be willing to comply with all study procedures and protocols;
- Be able to give written consent themselves;
- Be able to read and understand English;
- Be willing to sign the “Authorization for Release of Personal Health Information and Use of Personally Unidentified Study Data for Research” form; and,
- Understand that the data would only be used for research.

Exclusion Criteria for the calibration:

- Show evidence of extremely poor oral hygiene (made scoring extremely unreliable);
- Have been suffering from systemic diseases, significant past or medical history (i.e. severe/uncontrolled diabetes, HIV, heart conditions that require antibiotic prophylaxis, etc.); and,
- Present with other conditions that may have decreased the likelihood of their adhering to the study protocol.

All participating subjects gave informed written consent ahead of the calibration sessions.

##### Calibration procedures - data recording

**Fig. 1:**
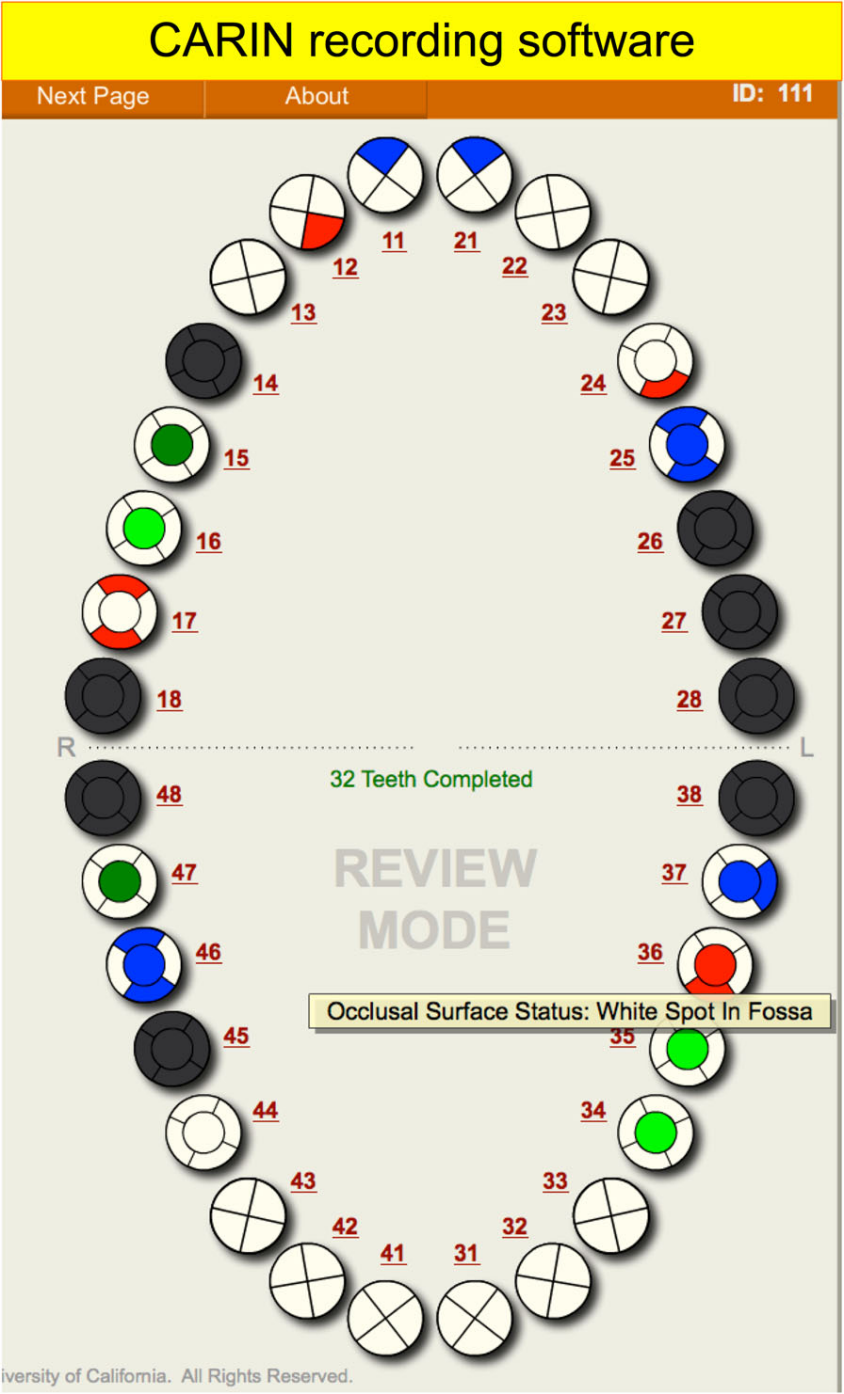
Typical charting in CARIN

The actual calibration with the gold standard examiner began after the training workshop was completed. For all calibration examinations, a data recorder who was experienced with the examination sequence, fully versed in direct data entry onto the laptop and software being used, and fully knowledgeable regarding the details of the specific clinical data elements and the recording protocol overall was present. Whenever possible, one data recorder would work exclusively with one dentist examiner for all exams during the calibration session to reduce recorder-based variability. The data recorder would ask for clarification from the examiner for any decision that was unclear or did not follow the coding of the data entry program. For data recording, CARIN software - CAries Research INstrument Software Package was used [19]. Fig. 1 demonstrates a typical charting in CARIN.

##### Minimum requirements of inter-examiner reliability for CAMBRA-PBRN examiners

In order to be certified as a successfully trained and calibrated examiner for the CAMBRA-PBRN study, each examiner had to meet a minimal level of inter-examiner reliability with the gold standard examiner, as assessed by the kappa statistics for the different levels of disease. Specifically, for DMFS, examiners had to reach kappa values of 0.75 or greater (adapted from [18]). For ICDAS-1 and 2, non-cavitated lesions evaluations, each examiner had to reach surface level kappa values of 0.40 or greater [18].

For the calibration, each dentist examined a minimum of 13 subjects whose exams were compared to those of the gold standard examiner. After the completion of these initial calibration examinations of an average of 13 subjects, which typically lasted about a half day, inter-examiner reliability was assessed in comparison to the gold standard examiners. If an individual examiner met the above stated standards on all levels of disease, he or she could be certified as standardized. If not, an additional set of subjects had to be seen until reliability was adequate for each level of disease.

### Examination criteria

#### Determination of teeth present

A tooth was considered present (code P) if any of its clinical crown projected through the gingival tissue. A tooth was scored as missing if the entire tooth (including roots) was not present in the mouth (code M).

#### Criteria for evaluating DMF scores (for all teeth and tooth surfaces)

A tooth surface was scored as decayed (code C) only if there was localized enamel breakdown due to carious demineralization of tooth structure. This code applied only to tooth surfaces that presented with a cavitated lesion. When a lesion on a posterior or anterior tooth extended beyond the line angle or marginal ridge onto another surface, then that other surface was also scored as code C.

If a restoration was present on the tooth surface, regardless of the restorative material used, that surface was scored as filled (code F). Sealants were scored as filled (code F). In the case of a multi-surface restoration present on a **posterior** tooth, the restoration needed to extend at least 1 mm beyond the line angle before it was considered to involve the adjacent surface. However, on a multi-surface proximal restoration on an **anterior** tooth, the adjacent labial or lingual surface would not be considered to be involved unless its margin extended **at least one-third** into these surfaces. The reason for this criterion was that tooth structure on surfaces adjacent to the primary carious lesion would most likely have been removed to provide access for the restoration of a proximal lesion on anterior teeth, and not necessarily because they were carious.

If a tooth surface was present and there was no evidence of cavitation or restorations on this surface, it could be scored as sound (code 0). White or discolored areas of the tooth surface due to hypoplasia, fluorosis, or staining were also scored as sound. Areas of enamel loss due to erosion or attrition or abfractions were scored as sound, as long as no enamel or dentin breakdown due to demineralization was present. However, if no part of a particular tooth surface was evident above the gingiva, then that surface was scored as unerupted (code U).

Incisal edges of anterior teeth were not considered separate surfaces. In the case that a lesion or restoration was confined solely to the incisal edge, its score was assigned to the nearest adjacent surface.

When a tooth was both filled and had a cavitated caries lesion, or when a filling presented with recurrent decay (cavitated), the tooth surface was scored as decayed (code C). Cavitated caries lesions took precedence over restorations. In the case of a restoration and non-cavitated decay (codes 1 or 2 – see below) present on the same tooth surface, the surface was scored as filled (code F), as restorations took precedence over non-cavitated decay.

Temporary restorations were scored in the same way as permanent restorations (code F). Fractured teeth were scored as sound (code 0) in the absence of caries. Fractured restorations were scored as intact (code F) unless there was an existing cavitated caries lesion present on that same surface. If a cavitated caries lesion was found within or adjacent to the margins of a fractured restoration, the cavitated caries lesion was scored only on the surfaces involved (code C).

Similarly, missing restorations were scored as intact teeth (code 0), unless there was a cavitated caries lesion present on that particular surface. If a cavitated caries lesion was found within or adjacent to the margins of a missing restoration, cavitated caries lesions were scored only on those surfaces involved (code C). When the tooth crown extensively damaged by caries and only the roots remained, all surfaces were scored as caries (code C).

#### Criteria for evaluating ICDAS scores (for eligible occlusal surfaces of posterior teeth only)

**Fig. 2:**
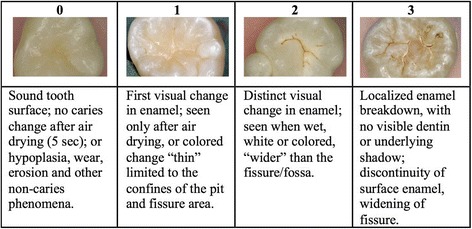
ICDAS scoring criteria for healthy (score 0), non-cavitated lesions (score 1 and 2) and first representation of a cavitated lesion (score 3)

After evaluating each tooth carefully for the DMF score, examiners were required to return to each posterior tooth (excluding 3rd molars), **which had occlusal surfaces that did not present with cavitated lesions or fillings**. The ICDAS scoring criteria (Fig. 2) for only these eligible occlusal surfaces were:

### ICDAS 0 - *Sound tooth surface*

No caries change was evident in pits and fissures of the occlusal surface after air-drying it for 5 s. Hypoplasia, wear, erosion and other non-caries phenomena were considered sound.

### ICDAS 1 - *First visual change in enamel*

Whitish-yellowish or brownish discoloration due to mineral loss caused by caries was detected within the confines of the pit and fissures of the occlusal surface. If there was generalized, uniform discoloration of all pits and fissures, then that discoloration was most likely general stains and would be scored a “0” instead.

### ICDAS 2- *Distinct visual change in enamel*

Whitish-yellowish or brownish demineralization of tooth enamel that was wider than the confines of the fossa. This discoloration should be going “up the slopes” of the fissures. In addition to receiving training on study participants, examiners were also trained on extracted teeth, which presented with ICDAS 0, 1, 2 lesions and first cavitations (up to 5 samples of each score).

Examiners knew that tactile examination with an explorer was not part of the CAMBRA-PBRN study protocol and should not be used to assess the tooth or surface status during the calibration. Examination for caries was focused on individual teeth and the condition observed on the tooth was scored independently of what was seen elsewhere in the mouth. However, previously difficult classifications became more clear after completing the exam. In these instances, it was acceptable to go back and reconsider certain scores based on knowledge that had become apparent after a thorough examination. Finally, the examiners were repeatedly advised that, when in doubt, they were to record the surface as the less severe, less involved condition.

### Statistical analyses

To calculate inter-examiner reliability, the dental status charting with existing restorations and the caries status (sound, non-cavitated lesions and DMFS scores) of the dentition determined by the examiners were compared to the “gold standard” findings. The inter-examiner reliability was calculated as kappa [20] (unweighted kappa). For the interpretation of numeric kappa values in this publication, the strength of agreement will be also described as ‘poor’ , ‘fair’ , ‘moderate’ , ‘good’ and ‘very good’ [21].

## Results

Sixty-one patients were recruited for the calibration sessions. These were 33 male and 28 female subjects with an average age of 38 ± 15 years (Mean ± Standard Deviation [SD]) and an age range from 12 to 63 years. These patients were seen in 94 patient encounters over the course of all six calibration sessions.

Thirty dentists (13 female, 17 male) were calibrated to the single gold standard examiner (BJ) during the 6 calibration sessions. Kappa statistics were used to determine inter-examiner reliability on an average of 13 or more patients per examiner. Each examiner scored between 1036 and 2220 tooth surfaces with an average of 1854 ± 252 (Mean ± SD) surfaces per examiner.

For each calibration session, 96.1% to 100% of patients had a DFS > 0. The minimum DS was 1.6 ± 1.6 (Mean ± SD) and the maximum DS score was 6.3 ± 4.5, while the minimum DFS score was 23.6 ± 17.6 and the maximum DFS was 27.9 ± 16.2.

The kappa values of the participating dentists compared to the gold standard examiner ranged from 0.75 to 0.90, with an average kappa of 0.84 ± 0.03 (Mean ± SD), which is considered as ‘very good’ agreement with the gold standard. 90% of the examiners achieved overall kappa values above 0.8 (‘very good’ agreement). These kappa values included the agreements in all scoring criteria – presence of teeth, fillings, sound and non-cavitated and cavitated lesions. After excluding ICDAS 1 and 2 scores (non-cavitated lesions) from the kappa calculation, the inter-examiner reliability between the examiners and the gold standard increased to 0.86 ± 0.04. A similar kappa value with 0.86 ± 0.03 was achieved when ICDAS 1 and 2 scores were merged into “sound.” When the presence of non-cavitated lesions versus absence of non-cavitated lesions on occlusal surfaces of non-filled molars and bicuspids was separately considered, the kappa value was only 0.55 ± 0.15, the examiners were only in ‘moderate’ agreement with the gold standard.

All dentists reached sufficient inter-examiner reliability values with regards to the gold standard to proceed into the main study. Two examiners needed to evaluate a second set of subjects to achieve sufficient inter-examiner agreements with the gold standard. One dentist discontinued at the end of the calibration study due to scheduling conflicts.

## Discussion

PBRNs were designed to offer advantages to both research and quality improvement [13, 22]. PBRN studies have the potential to quickly move scientific advances right into daily practice and support sharing of information between practitioners [23]. With those advantages in mind the new CAMBRA-Practice Based Research Network study (CAMBRA-PBRN) was planned to prove that CAMBRA can be successfully implemented in dental practices outside of the typical university setting. For that reason, 30 dentists in the San Francisco Bay Area were approached to participate in the CAMBRA-PBRN study.

This calibration study was performed so that all participating dentists could score oral conditions in a given study subject in a consistent manner [24] and to assure comparability between contributing offices. During the calibration meetings, dentists were trained and standardized in scoring decayed, missing, and filled surfaces (DMFS) [16, 17]) in the dentition. Since non-cavitated caries lesions in enamel and dentin can be managed by remineralization without restorative intervention [25, 26], the dentists were also calibrated in classifying caries lesions at a non-cavitated stage [15]. This would allow the participating dentists to evaluate the success of non-invasive CAMBRA measures applied in the main study [27]. Accordingly, the dentists were specifically trained to differentiate between sound (ICDAS 0), non-cavitated caries lesions (ICDAS 1 or 2) (International Caries Detection and Assessment System) [14, 15] and cavitated caries lesions.

In addition to demonstrating preventive effects when including non-cavitated lesions as study lesions into the field of randomized clinical caries trials, it was demonstrated that detecting differences in treatment effect over a shorter time period was possible. If clinical visual diagnostic criteria can be applied, which include lesions confined to enamel, compared to using criteria relying on only on later stages of caries already extending into dentin, clinical trials could be abbreviated [28]. This is based on the assumption that many therapies directed at remineralization should protect enamel similarly across lesion severities from initiation to near cavitation. Imrey and coworkers in 2004 stated ‘if this is so, and if acceptable reproducibility and predictive validity can be demonstrated for a diagnostic of acceptable cost, then clinical trials of agents to prevent cavitation can become more efficient by the use of outcome indices that reflect, in addition to cavitation, the expansion and regression of non-cavitated lesions’ [29]. An International Consensus Workshop on Caries Clinical Trials (ICW-CCT) held in 2002 stated that ‘the understanding of the caries process has progressed,’ and ‘for future clinical trials, recording only cavitated lesions as an outcome measure is becoming outmoded’ [30]. Furthermore, not only the time duration of a clinical trail might be reduced. The number of subjects to be included in a trial to demonstrate a significant effect of an intervention, when changes in the continuum of the caries process recorded as arrestment or reversal of early stages of mineral loss are documented may also be decreased. In a clinical trial, Chester et al. detected after 12 months significant differences in efficacy between 1000- and 2500-ppm-fluoride dentifrices when the D_1_ (enamel and dentin) threshold was included in the assessment. This study confirmed the validity of an abbreviated trial protocol [28].

In the CAMBRA-PBRN calibration study presented here, the 30 dental practitioner examiners were trained and tested to score the conventional DMFS index and additionally non-cavitated lesions at the ICDAS level score 1 and 2, respectively. With an average kappa of 0.84 ± 0.03 (Mean ± SD), the inter-examiner reliability compared to the gold standard was considered as ‘very good’. This scoring level included the scoring of cavitated, as well as, non-cavitated lesions. When the scoring for non-cavitated lesions alone was compared between the gold standard and the examiners, the reliability level decreased to a moderate level with a kappa value of 0.55 ± 0.15. The moderate kappa value for non-cavitated lesions indicates the difficulty of agreement in clinically differentiating initial demineralization effects, thus showing the limitations of the calibration effort. Incorporation of the more detailed ICDAS caries staging for future studies might require additional time and clinical education [31].

In this calibration study, the inter-examiner reliability values achieved between the gold-standard examiner and examiner-trainees with regards to the DMFS index were at least as high as typically found in the literature [32]. When the ICDAS components score 1 and 2, as non-cavitated lesions, were calculated separately, the kappa values also appeared to be at a similar level as reported by others. Ekstrand et al. [33] stated for visual caries examination scores, equivalent to the ICDAS scores, on 120 extracted teeth performed by 3 examiners, comparable slightly lower kappa coefficients ranging between 0.54 and 0.69 for the inter-examiner reliability. Fyffe et al. [34] trained 20 crews of Community Dental Officers teamed up with a dental nurse for the United Kingdom’s national dental health services on recording dental caries. When assessing levels of enamel caries including caries at the D_1_ (enamel and dentin) diagnostic threshold, which could benefit from preventive care, as well as dentinal caries requiring restorative treatment, they reported mean inter-examiner kappa coefficient ranges for novice examiners that were similar to those in our study between 0.47 and 0.53 for non-cavitated carious lesions and 0.64 and 0.66 for cavitated lesions [34]. In a cohort study by Ismail et al. conducted by the Detroit Center for Research on Oral Health Disparities, the inter-examiner reliability of six examiners classifying tooth surfaces were relatively high using ICDAS caries criteria. They ranged between good to excellent with kappa coefficients between 0.59 and 0.82 [15].

There are huge differences in the requirements of studies for calibration. In the calibration procedures for the prevention trials of the Early Childhood Caries Collaborating Centers, only 3 examiners were calibrated and re-calibrated requiring very high inter-examiner reliability levels. Due to challenges in caries examination of pre-school children related to lack of cooperation, frequent movement, and difficulty in keeping teeth dry, caries lesions were only differentiated in non-cavitated and cavitated. The training and calibration was done partially using electronic training materials and clinical examination of children of ages under 3 years as well as above. Similar to our calibration program, study examiners saw at least 13 subjects per calibration session [18]. In contrast, in the Prevention of Adult Caries Study (PACS), regular community dentists working in multiple centers on the East and West coasts of the US were calibrated. The PACS study also used non-cavitated lesions for the net outcome caries increment, but they collapsed different ICDAS scores into single codes of non-cavitated versus cavitated lesions [35] to facilitate quicker routine examinations of the study patients.

## Conclusions

Thirty dentists, potential participants in a CAMBRA-PBRN-study, were trained and calibrated successfully in DMFS and ICDAS-scoring. The inter-examiner reliability to a gold standard was high. The high inter-examiner reliability results have shown thus far that dentists who work in primarily non-research based practices can be effectively trained, standardized and calibrated in data collection, based on specific guidelines created to anticipate potential research study scenarios. However, separate reliability for assessment of non-cavitated lesions, as in other studies, was lower.

## Abbreviations

CAMBRA: Caries management by risk assessment
CARIN: Caries research instrument software package
CDA: California dental association
DMFS: Number of decayed, missed or restored tooth surfaces
FQHC: Federally qualified health center
ICDAS: International caries detection and assessment system
IRB: Investigational review board
SD: Standard deviation
UCSF: University of California San Francisco
US: United States
